# Combinations of bio-active dietary constituents affect human white adipocyte function in-vitro

**DOI:** 10.1186/s12986-016-0143-5

**Published:** 2016-11-21

**Authors:** Ines Warnke, Johan W. E. Jocken, Rotraut Schoop, Christine Toepfer, Regina Goralczyk, Joseph Schwager

**Affiliations:** 1 DSM Nutritional Products Ltd., Department of Human Nutrition and Health, CH-4002 Basel, Switzerland; 2 Department of Human Biology, NUTRIM School of Nutrition and Translational Research in Metabolism, Maastricht University Medical Center+, Maastricht, The Netherlands

**Keywords:** Primary human adipocyte function, Lipid accumulation, Adipokine secretion, Adipogenic/lipogenic genes, Lipolytic genes, Lycopene, Resveratrol, EPA, Combinations of bio-actives

## Abstract

**Background:**

Specific bio-active dietary compounds modulate numerous metabolic processes in adipose tissue (AT), including pre-adipocyte proliferation and differentiation. AT dysfunction, rather than an increased fat mass *per se*, is strongly associated with the development of insulin resistance and is characterized by impaired adipogenesis, hypertrophic adipocytes, inflammation, and impairments in substrate metabolism. A better understanding of mechanisms underlying AT dysfunction may provide new strategies for the treatment of obesity-associated metabolic diseases. Here we evaluated the role of (all-E)-lycopene (Lyc), eicosapentaenoic acid (EPA) or *trans*-resveratrol (Res) and combinations thereof on human white adipocyte function.

**Methods:**

In-vitro differentiating human pre-adipocytes were treated with EPA, Lyc and Res or their combinations for 14 days. The effects on intracellular lipid droplet (LD) accumulation, secreted anti- and pro-inflammatory cyto-/adipokines (e.g. adiponectin, IL-6, IL-8/CXCL-8 and MCP-1/CCL2) and on gene expression of markers of adipocyte differentiation and substrate metabolism (e.g. PPAR-gamma, C/EBP-alpha, GLUT-4, FAS, ATGL, HSL, and PLIN-1) were measured by fluorescent microscopy (Cellomics™), multi-parametric LiquiChip® technology and quantitative RT-PCR, respectively.

**Results:**

Treatment of differentiating adipocytes for 14 days with the combination of Lyc/Res and EPA/Res resulted in significantly inhibited LD formation (~ -25 and -20%, respectively) compared to the effects of the single compounds. These morphological changes were accompanied by increased mRNA levels of the adipogenic marker PPAR-gamma and the lipase ATGL and by decreased expression levels of lipogenic markers (LPL, FAS, GLUT-4) and the LD-covering protein PLIN-1. In addition, a blunted adipocyte secretion of pro-inflammatory cytokines (IL-6 and MCP-1) and adiponectin was observed following treatment with these compounds.

**Conclusion:**

The combination of the dietary bio-actives Lyc and EPA with Res might influence adipocyte function by affecting the balance between adipogenic, lipogenic and lipolytic gene expression, resulting in a reduced LD storage and a less inflammatory secretion profile. Taken together, our results indicate that combinations of dietary compounds may be beneficial for the prevention and treatment of metabolic disorders via effects on human white adipocyte function.

**Electronic supplementary material:**

The online version of this article (doi:10.1186/s12986-016-0143-5) contains supplementary material, which is available to authorized users.

## Background

Over the last decade research focusing on adipose tissue (AT) biology and function enormously advanced due to the sustained increase in obesity prevalence [1]. AT dysfunction, characterized by impaired adipogenesis, hypertrophic adipocytes, inflammation, and impairments in lipid and glucose metabolism, rather than an increased body fat mass *per se*, is strongly associated with the development of insulin resistance [2]. A better understanding of mechanisms causing or maintaining AT dysfunction may provide novel and improved strategies for the treatment of obesity-associated metabolic diseases.

The major role of AT is storage and release of fatty acids (FAs) depending on energy intake and expenditure. FAs are stored in the form of triacylglycerides (TAGs) in intracellular lipid droplets (LDs) and released by lipolysis, the hydrolysis of TAGs into free FAs and glycerol via the action of intracellular lipases (including hormone sensitive lipase (HSL) and adipose triglyceride lipase (ATGL)). This storage and removal capacity (lipid turn-over) of AT is regulated by a tight alignment between adipogenic differentiation, lipogenesis and lipolysis [3, 4], which has been shown to be impaired in obesity and may modulate whole-body insulin sensitivity [5, 6]. The differentiation of pre-adipocytes into mature adipocytes is controlled by a complex transcriptional cascade involving peroxisome proliferator-activated receptor gamma (PPAR-gamma) and CCAAT/enhancer binding protein alpha (C/EBP-alpha) (reviewed in [7]). Furthermore, lipid-storing adipocytes are enclosed by adipose-derived stromal cells including pre-adipocytes, endothelial and hematopoietic cells, and immune cells (e.g. macrophages [8]). Hence, AT is not only an energy storage tissue but also an active endocrine organ, producing and secreting an abundance of specific mediators (for review see [9]). Pro-inflammatory cytokines and chemokines are increasingly secreted by AT cells of obese individuals [10], resulting in a state of ‘low-grade inflammation’ [11], which affects local adipose metabolism, systemic inflammation and insulin sensitivity.

Overall dietary quality and specifically diets high in bio-active constituents may have beneficial clinical effects on metabolic processes, by altering AT function. A number of natural compounds, such as plant-derived polyphenols, carotenoids and polyunsaturated fatty acids (PUFAs), or their metabolic derivatives have been tested for their impact on adipocyte differentiation and metabolism in several in-vitro and in-vivo murine models [12–14]. However, human data are scarce and mainly the action of individual compounds have been tested [15, 16]. Several studies using pre-adipocytes, mostly from murine and less frequent from human origin, have demonstrated that the polyphenol Res and the n-3 PUFA EPA are potent modulators of adipocyte function (for review see [17, 18], respectively). However, distinct biological activities of lycopene or its metabolites in human adipocyte function remain to be elucidated. Here, we investigated the effects of individual and combinations of bio-active dietary constituents including (all-E)-lycopene (Lyc), eicosapentaenoic acid (EPA) and *trans*-resveratrol (Res) on lipid accumulation, adipogenic, lipogenic and lipolytic gene expression and cyto-/adipokine secretion in in-vitro differentiating (14 days) primary human white adipocytes.

## Methods

### Cell culture

All cell culture reagents were obtained from Life Technologies (Karlsbad, CA, US). Unless otherwise stated, chemicals were purchased from Sigma-Aldrich (St. Louis, MO, US). Individual subcutaneous primary human pre-adipocytes (HPAd 1375 and 1377) were obtained from Cell Application, Inc. (San Diego, CA, US) whereas a multi-donor vial, termed super lot (SL0035), was purchased from Zen-Bio, Inc. (North Carolina, US). Available donor characteristics are indicated in Additional file 1: Table S1. Pre-adipocytes were maintained in growth medium (GM): DMEM/Ham’s F-12 (1:1, v/v) complemented with 10% FCS, 1% pen/strep (v/v), 1% HEPES pH 7.4, 0.2% amphotericin B, and 2.5 ng/ml recombinant basic fibroblast growth factor (bFGF). GM was changed every 2–3 days. Cells were passaged when reaching ~80% confluence and used for experiments between passage 3 and 7. For experiments, 6000 cells/cm^2^ were incubated (37 °C, 5–8% CO_2_, relative humidity of 85%) on collagen-I-coated 24-well plates in GM for ~5 days. For adipogenesis confluent HPAd were cultured for 14 days in differentiation medium (DM): DMEM/Ham’s F-12 supplemented with 5% FCS, 1% pen/strep, 1.5% HEPES pH 7.4, 0.2% amphotericin B, 17 μM calcium-pantothenate, 33 μM biotin, 0.5 μM recombinant human insulin and 100 μM rosiglitazone. After 3 days of differentiation 0.5 μM dexamethasone and 250 μM isobutylmethylxanthine (IBMX) were omitted from the medium.

For the non-differentiation control (CTRL) and differentiation control (Diff CTRL) pre-adipocytes were cultured in medium containing only vehicle (i.e. dimethylsulfoxide (DMSO), tetrahydrofurane (THF)) and insulin or in complete DM, respectively. For treatment purposes DM was supplemented with different doses (0.5–25 μM) of the test compounds EPA, Res and Lyc (DSM Nutritional Products Ltd. Basel, Switzerland)) for the total 14 day differentiation period (see Additional file 2: Figure S1). Lyc was dissolved in THF and all other substances in DMSO. To investigate possible amplifying effects, Res (1 and 25 μM) was also combined with Lyc (0.5 and 2 μM) or EPA (1 and 25 μM), respectively. Treatments were performed in triplicate. Final vehicle concentrations were adjusted to 0.2% DMSO and 0.1% THF in all cultures. Media with or without bio-active compounds were renewed every 3 to 5 days.

### Lipid droplet quantification (ArrayScan)

Cellular LDs were quantified adapting the Cellomics™ assay described previously [19] applying the Thermo Scientific™ ArrayScan™ VTI High Content Reader (Thermo Fisher Scientific, Waltham, MA, US). Briefly, differentiated adipocytes (day 14) were fixed, stained with the fluorescent dyes Hoechst 33342 (nuclei) and BODIPY® 493/503 (LDs; Life Technologies) followed by quantification of accumulated LDs with the SpotDetector® V2 algorithm. Adjusted protocol parameters are listed in Additional file 3: Table S2. For analysis 100 fields per well were scanned and data of each channel were reported on a “per field” basis. The nuclei related features Spot Count (= LD-number), Spot Total Area (= LD-area) and Spot Total Intensity (= LD-intensity), describing the differentiation status of treated adipocytes, were calculated as percent of Diff CTRL per plate.

### Gene expression analysis (TaqMan™)

Total RNA was isolated from cells at day 8 of treatment (RNeasy® 96 Kits; Qiagen, Hilden, Germany). Primers and probes were designed using the Primer Express software (Applied Biosystems, Foster City, CA, US) and synthesised by Sigma Genosys (St. Louis, MO, US) (Additional file 4: Table S3). Quantitative TaqMan™ RT-PCR was performed on first strand cDNA (Omniscript® RT Kit; Qiagen) as detailed previously [19] utilizing an ABI-PRISM® 7900 HT Sequence Detection System. mRNA abundance was calculated using the comparative C_T_ method: ΔC_T_ = C_T_ [*gene of interest*] – C_T_ [*endogenous control*] and ΔΔC_T_ = ΔC_T_ [*Diff CTRL cells*] - ΔC_T_ [*treated cells*]. The fold expression for the gene of interest was expressed as ${2}^{-\Delta \Delta {\mathrm{C}}_{\mathrm{T}}}$.

### Adipokine and cytokine secretion (Luminex)

Supernatants of differentiating adipocytes were collected at day 8 (after 5 days conditioning, day 4–8) of the treatment period and stored at -80 °C till analysis. MILLIPLEX MAP Human Adipocyte Panel (Cat#HADCYT-61 K) kits were purchased from Millipore (Billerica, Massachusetts, USA) and used according to the manufacture’s protocol on the LiquiChip® Workstation IS 200 (Luminex technology; Qiagen, Hilden, Germany). Detected molecules were: adiponectin (Adipo), hepatic growth factor (HGF), interleukin (IL)-6, IL-8/CXCL8, monocyte chemoattractant protein (MCP)-1/CCL2 and plasminogen activator inhibitor (PAI)-1 (active, serpin E1). Measurements were run in triplicates and final concentrations were normalized by nucleus count per well. Data evaluation was performed with the LiquiChip® Analyser software from Qiagen.

### Statistical analysis

In brief, data points from repetitive experiments conducted with pre-adipocytes from the same donor were set relative to the corresponding Diff CTRL mean (=100%) and averaged. Subsequently all relative values from the different donors and super lot (Additional file 1) were used for calculating the overall mean ± SEM. Statistical significance of the mean differences between treatment and Diff CRTL was tested by a linear mixed model or Student's *t*-test. *P* values <0.05 were considered significant. ArrayScan™ results and cytokine concentrations are shown as relative mean ± SEM. Gene expression data are expressed as fold change (FC) ± error (based on SEM). For details of the statistical analysis applied to the three data sets see Additional file 5.

## Results

### Combinations of bio-active dietary constituents inhibited lipid accumulation

**Fig. 1:**
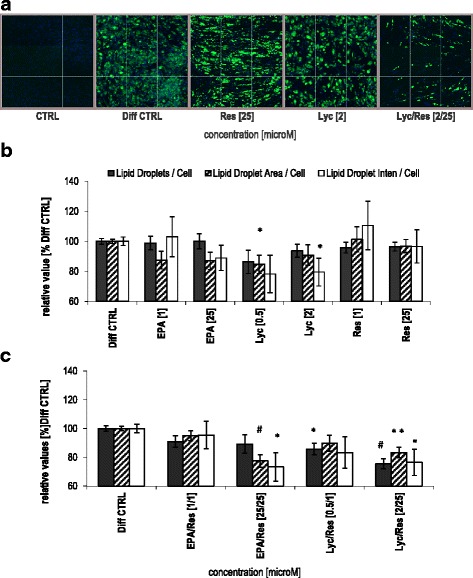
Effects of Lyc, Res and EPA on lipid droplet parameters in differentiated human adipocytes. Primary human pre-adipocytes (HPAd) were differentiated for 14 days in the presence of bio-active compounds. **a** Representative fluorescent images of cells after growth in non-differentiation medium (CTRL) and differentiation-only medium in the absence (Diff CTRL) or presence of resveratrol (Res), lycopene (Lyc) and Lyc/Res at high concentrations. **b** Quantification of the effects of Lyc, Res and eicosapentaenoic acid (EPA) alone and **c** of the combinations of EPA/Res and Lyc/Res on lipid droplet features. Shown are three parameters (Lipid Droplets/Cell, Lipid Droplet Area/Cell and Lipid Droplet Intensity/Cell) that quantify lipid accumulation in adipocytes, as compared to Diff CTRL set as 100%. Data are represented as overall mean ± SEM (7 donors, n ≥ 6). (*) *p* < 0.05, (**) *p* < 0.01, (#) *p* < 0.001 (versus Diff CTRL, linear mixed model)

To determine effects of bio-active dietary constituents on lipid accumulation in adipocytes, pre-adipocytes were differentiated for 14 days in the presence or absence of the compounds. Subsequently, LD accretion in the mature adipocytes was assessed by fluorescent microscopy (Fig. 1a). Lyc alone decreased the LD-area by 15% at 0.5 μM (*p* = 0.021) and the LD-intensity by 20% at 2 μM (*p* < 0.05), while Res and EPA alone did not affect lipid accumulation (Fig. 1b) compared to Diff CTRL. However, the combination Lyc/Res substantially reduced the adipocyte lipid content (represented by shown LD-parameters). Lyc/Res at 2/25 μM, significantly inhibited LD-number, -area and -intensity by 25% (*p* < 0.001), 17% (*p* < 0.01) and 23% (*p* = 0.044), respectively (Fig. 1a + c, right), in comparison with the Diff CTRL. In addition, combined treatment with EPA/Res at 25/25 μM reduced LD-area and -intensity by 22% (*p* < 0.001) and 26% (*p* = 0.011), respectively, whereas the LD-number was slightly attenuated (Fig. 1c). Together, these data indicate that the combination of Lyc/Res and EPA/Res significantly reduced the adipocyte lipid content compared to Diff CTRL.

### Dietary bio-actives affected adipogenic, lipogenic and lipolytic gene expression

**Fig. 2:**
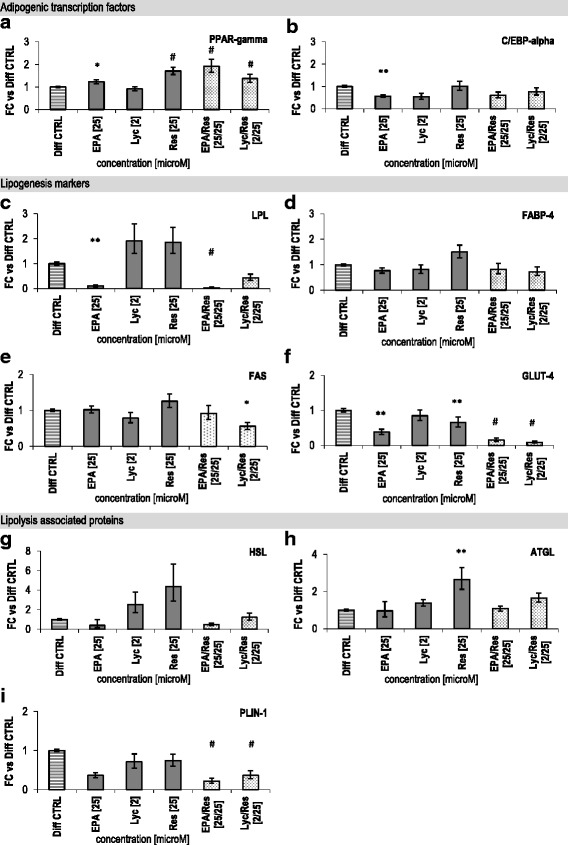
Effects of bio-active compounds on adipogenic, lipogenic and lipolytic gene expression markers. Displayed are the effects of EPA, Lyc and Res or combinations thereof on gene expression after 8 days treatment of HPAd. mRNA levels of adipogenic transcription factors PPAR-gamma **a** and C/EBP-alpha **b**, lipogenesis markers LPL **c**, FABP-4 **d**, FAS **e** and GLUT-4 **f** and lipolytic markers HSL **g**, ATGL **h** and PLIN-1 **i** were determined by quantitative RT-PCR. Data are shown as crude fold change (FC) ± error (based on SEM, 2 donors, 1 super lot, n ≥ 2), compared to Diff CTRL set as 1. (*) *p* < 0.05, (**) *p* < 0.01, (#) *p* < 0.001 (versus Diff CTRL, linear mixed model). CV for dCT values: <5% for all genes

In order to investigate the possible underlying mechanism for this attenuated lipid accumulation targeted quantitative RT-PCR analysis was performed at day 8 of differentiation, a time point intermediate between undifferentiated and differentiated adipocytes. The combinations EPA/Res and Lyc/Res at high concentrations increased the expression of the adipogenic master regulator, PPAR-gamma, by 1.9-fold and 1.4-fold (*p* < 0.001), respectively, compared with Diff CTRL (Fig. 2a). Lyc/Res reduced mRNA levels of the lipogenic genes FAS (fatty acid synthase) and GLUT-4 (glucose transporter type 4 /SLC2A4) significantly (Fig. 2e + f, *p* < 0.05). In addition, the combination of EPA/Res strongly blunted the lipogenic markers LPL (lipoprotein lipase) and GLUT-4 mRNA (0.03 and 0.15-fold, respectively; Fig. 2c + f, *p* < 0.001). Both combinations did not affect FABP-4 (cytosolic fatty acid binding protein 4) expression (Fig. 2d). Res alone at 25 μM moderately enhanced the expression of PPAR-gamma to 1.7-fold (*p* < 0.001) and reduced GLUT-4 expression 0.7-fold (*p* = 0.007) compared to Diff CTRL. The expression of C/EBP-alpha, LPL and GLUT-4 was diminished to 0.5-fold, 0.1-fold and 0.4-fold, respectively, when pre-adipocytes were differentiated in the presence of 25 μM EPA (Fig. 2b, c + f, *p* < 0.01). In contrast, Lyc alone (at 2 μM) did not affect adipogenic and lipogenic gene expression (Fig. 2a-f).

The mRNA levels of the major lipases HSL and ATGL were neither significantly affected by Lyc and EPA nor by the combinations, although Res alone increased ATGL expression 2.6-fold (*p* = 0.006; Fig. 2g + h) in comparison to Diff CTRL. However, combining these bio-active compounds significantly diminished the expression of the LD-covering protein PLIN-1 (perilipin 1; Fig. 2i, EPA/Res: 0.2-fold and Lyc/Res: 0.4-fold, *p* < 0.001). A comparable effect on the expression of lipogenic and lipolytic genes was observed at day 14 for both combinations (Additional file 6: Figure S2). Altogether, these results demonstrate that combining of Lyc/Res and EPA/Res affects the balance between adipogenic, lipogenic and lipolytic gene expression in white human adipocytes.

### Bio-active compounds attenuated secretion of pro-inflammatory cytokines

**Fig. 3:**
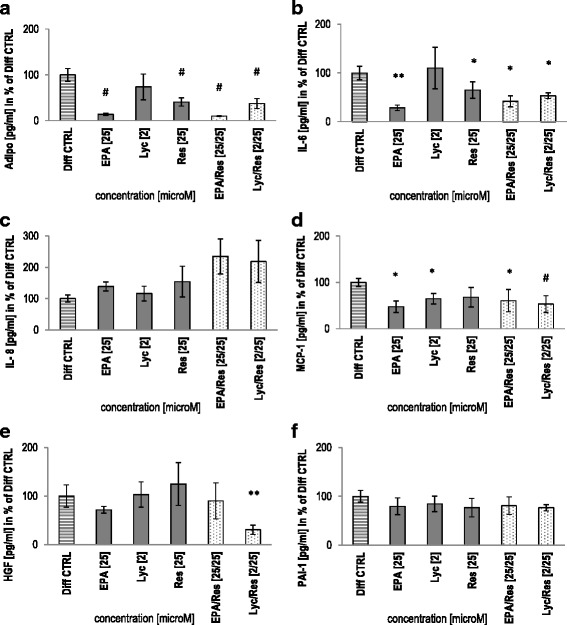
Adipokine and cytokine secretion of adipocytes treated with bio-active compounds. Supernatants of HPAd, cultured in the presence or absence of EPA, Lyc and Res or combinations thereof, were collected at day 8 of differentiation and subsequently analysed on the LiquiChip® workstation. The accumulation of the following adipokines: adiponectin (Adipo, **a**), interleukin-6 (IL-6, **b**) and 8 (IL-8, **c**), monocyte chemotactic protein 1 (MCP-1, **d**), hepatic growth factor (HGF, **e**) and plasminogen activator inhibitor-1 (PAI-1, **f**) was measured in media after 5 days conditioning (day 4–8), as compared to Diff CTRL set as 100%. Data are shown for three independent experimental series as overall mean ± SEM. (*) *p* < 0.05, (**) *p* < 0.01, (#) *p* < 0.001 (versus Diff CTRL, linear mixed model)

Finally, we analysed the influence of dietary bio-actives on cyto-/adipokine secretion of differentiating human adipocytes. EPA alone and the combination EPA/Res strongly reduced the secretion of the insulin sensitizer Adipo by ~90% whereas Res and Lyc/Res blunted it by ~60% (Fig. 3a) compared to Diff CTRL. The combinations EPA/Res and Lyc/Res and EPA suppressed pro-inflammatory IL-6 secretion by 70%. Release of the pro-inflammatory cytokine MCP-1 was significantly reduced by more than 30% by all compounds and combinations except Res. Both combinations caused a similar secretion pattern for the above mentioned cyto-/adipokines at day 14 of differentiation (Additional file 6: Figure S2). Further, the secretion of angiogenic HGF was markedly diminished by Lyc/Res (70%, *p* < 0.01), whereas PAI-1 levels were not modified by the compounds (Fig. 3e + f) in comparison to the Diff CTRL.

Together, our data indicate that EPA and the combinations Lyc/Res and EPA/Res are able to suppress the secretion of pro-inflammatory markers, but concurrently attenuate the anti-inflammatory and insulin sensitizing adipocyte-specific hormone adiponectin.

## Discussion

Weight gain is accompanied by increased lipid storage in AT, altered gene expression and production of pro- and anti-inflammatory mediators [20]. In the present study, we examined the effect of the three abundantly consumed bio-actives Lyc, Res and EPA on human white adipocyte function and whether combining Res with EPA or Lyc has amplifying effects. Our in-vitro data demonstrate that combining Lyc with Res caused a significant reduction in LD-number, -area and -intensity in human adipocytes. This reduced lipid accumulation was accompanied by increased expression of adipogenic and reduced expression of lipogenic and lipolytic markers, and by an attenuated pro-inflammatory secretion profile (i.e. IL-6 and MCP-1).

Single bio-active dietary constituents are known to influence pre-adipocyte differentiation, proliferation and adipocyte function directly or indirectly [15–18]. Surprisingly, in our study Res and EPA treatment alone showed no significant effect on the LD accumulation in human adipocytes. This is contrary to the general reported findings in in-vitro and animal studies, which suggest that Res and EPA are capable of diminishing lipid storage in rodent adipocytes [21–24]. However, in human adipocytes lipid accumulation was either promoted or unaffected by EPA [25, 26], what seems in line with our observation after 14 days treatment. For Res, both enhancing and suppressive effects on lipid accumulation were described in murine 3T3-L1 cells and following a high fat diet (HFD) in mice [27, 28]. In human Simpson-Golabi-Behmel syndrome (SGBS) adipocytes anti-adipogenic effects of Res were reported for concentrations > 30 μM [29, 30], suggesting that Res at 25 μM (this study) might be at switch between its pro- and anti-adipogenic actions. The limited number of human clinical trials with Res or EPA supplementation showed controversial results regarding their effects on adipocyte size, function [31, 32] and adipose inflammation [33, 34], possible due to variation in population, dose and duration of supplementation. Together, these data indicate that further research is necessary to elucidate the exact effect of Res and EPA on adipogenic potential of human (pre-) adipocytes, under the consideration that the usage of primary white adipocytes can affect the experimental outcomes due to intrinsic donor characteristics [35].

Supplementation with the carotenoid Lyc can increase its levels in human AT [36] and 3T3-L1 cells [37] and it is well studied for its anti-oxidative effects [38]. In addition, our results show for the first time that Lyc alone moderately but significantly reduces lipid accumulation in human adipocytes. This effect was also observed in C3H10 T1/2 adipocytes [19]. Conversely, Lyc or its metabolite apo-10’-lycopenoic acid (APO10LA) did not modulate AT mass in rodents [39] (following oral administration for 6 weeks) [40]. In contrast to the minor effect of single compounds, our data clearly demonstrate that the combination Lyc/Res has a stronger effect on LD accumulation, suggesting that Res enhances Lyc’s modest anti-adipogenic effect.

Although not a direct measure of lipid turn-over within adipocytes, investigating mRNA levels of genes involved in related pathways could provide possible underlying mechanisms for the amplifying effects observed with combinations of bio-actives. Res’s anti-adipogenic properties may be attributed to its effects on both master regulators of adipogenesis, PPAR-gamma and C/EBP-alpha, which are repressed via activation of AMP-activated protein kinase (AMPK) [41]. Interestingly, in the present study Res at 25 μM up-regulated PPAR-gamma expression and showed no effect on C/EBP-alpha mRNA in differentiating human adipocytes. This seems contrary to the reported down-regulation of PPAR-gamma and lipogenic markers in 3T3-L1 [41] and SGBS cells [29] at concentrations >25 μM and the accompanying reduction of fat pads in mice following 10 weeks of HFD feeding [42]. We hypothesize that in the present study the concentration of Res (25 μM) is insufficient to counteract the strong adipogenic effects of the PPAR-gamma agonist rosiglitazone [43], contained in the media at 100 μM during the complete differentiation period. This is further supported by results in differentiating SGBS cells, demonstrating no effect of 20 μM Res on PPAR-gamma expression even in the presence of a low rosiglitazone concentration (2 μM) [29].

PUFAs can act as PPAR agonists and display PPAR-independent gene control function [44]. Regarding EPA, a well-known n-3 PUFA, a reduction in stored LDs and a concomitant modification of adipogenic markers, e.g. a decrease of PPAR-gamma expression, was described in murine fat cells [19, 21]. However in line with our results, an upregulation of PPAR-gamma expression is described in isolated human adipocytes [45], suggesting species-specific effects of EPA on adipogenesis. Finally, Lyc is not presented as an adipogenic modulator [16], however some conflicting data exist regarding its impact on adipose gene expression. In accordance with our human data, in murine adipocytes, Lyc treatment did not affect adipogenic genes [39] but moderately decreased lipid deposition [19], whereas Lyc administration for 6 weeks decreased PPAR-gamma mRNA in AT of rats without changes in total fat mass [46].

In addition to adipogenesis, the balance between lipogenesis and lipolysis largely influences total lipid amount in human adipocytes. The combination of Lyc/Res and EPA/Res strongly suppressed the lipogenic markers GLUT-4, LPL and FAS in our human fat cells. However, for the single compounds conflicting effects on these markers were reported previously. Supporting our results, a reduction of GLUT-4 mRNA and unaffected levels of FAS mRNA were shown in 3T3-L1 cells after Res (20 μM) [47] and EPA (100 μM) [48] treatment, respectively. Whereas, opposing to our human data, GLUT-4 and LPL mRNA expression was up-regulated after treating murine adipocytes with Lyc (2 μM) [19] and EPA (100 μM) [21], respectively.

Furthermore, the lipases ATGL and HSL and the LD-covering protein PLIN-1 are key proteins in the intracellular lipolytic process, being tightly regulated by neuroendocrine signals (reviewed in [49]). Our results are in agreement with Lasa et al. [50], who reported that the mRNA level of ATGL but not HSL, were increased following treatment of mature human and murine adipocytes with 100 μM Res for 24 h, which was accompanied by an increased FFA release [50]. Interestingly, the mRNA of the dominant LD-covering protein PLIN-1 was not altered by the single constituents. In line, a recent in-vitro study in human adipocytes also demonstrated that EPA alone had no impact on PLIN-1 expression [51]. On the other hand, the combinations significantly suppressed PLIN-1 mRNA in our human adipocyte model, suggesting interactions between bio-active compounds which influence PLIN-1 expression. However, it needs to be investigated whether this translates into functional changes in the lipolytic potential of the adipocytes.

Together, the pronounced down-regulation of lipogenic (GLUT-4, LPL, FAS) and lipolytic genes (PLIN-1) by the combination of bio-active compounds (EPA and Lyc with Res) cannot be justified by additive effects of the single compounds, suggesting strong amplifying/synergistic interactions. Therefore, combining bio-active constituents facilitates the interplay of different direct and indirect transcriptional controlling mechanisms, which warrants further investigations.

Finally, we showed that the reduction in lipid accumulation following EPA/Res or Lyc/Res treatment was accompanied by a diminished release of the pro-inflammatory cytokines IL-6 and MCP-1. In line, a previous study, in different adipocyte models and in murine AT explants, demonstrated that pre-incubation with the anti-inflammatory nutrient Lyc (24 h, 2 μM) reduced the secretion of the pro-inflammatory cytokines IL-6 and MCP-1 induced by tumour necrosis factor (TNF)-alpha stimulation or HFD-feeding, respectively [52]. Additionally, a study with HFD-induced obese rats showed that Lyc supplementation increased plasma adiponectin levels and decreased the mRNA of MCP-1 and IL-6 in AT and their circulating protein levels [40]. Without stimulation of the inflammatory pathways (this study), Lyc only suppressed the secretion of the macrophage attracting chemokine MCP-1 [53] from human adipocytes. Furthermore, several studies indicate that treatment of mature SGBS and 3T3-L1 adipocytes with Res (100 μM, 48 h) [30, 54] and EPA (200 μM, 48 h) [55, 56], respectively, and supplementation of HFD-fed mice with both bio-actives (Res, 10 weeks [42]; EPA, 5 or 11 weeks [55, 56]) can reduce the expression and secretion of pro-inflammatory cytokines such as MCP-1, IL-6 and IL-8 in AT.

In addition, we observed a decrease of Adipo secretion with Res, EPA and the combinations. However, several groups reported an increase of the anti-inflammatory and insulin sensitizing adipokine adiponectin and a decrease of leptin expression and secretion after treatment of mature 3 T3-L1- and SGBS-adipocytes with Res (up to 200 μM) or its metabolites for 24 or 48 h, respectively [30, 57]. Similarly, EPA treatment (100 μM, 24 or 48 h) was also able to increase Adipo secretion from mature primary human adipocytes [26, 58]. Nevertheless, our findings are in line with recent data from Lorente-Cebrian et al. [59], who reported that 200 μM EPA (96 h) significantly decreased adiponectin gene expression and protein secretion in freshly isolated rat adipocytes [59]. Furthermore, we noticed a strong attenuation of the angiogenic protein HGF by the combination Lyc/Res. HGF is secreted from AT and its expression is elevated in the obese state allowing for remodelling of AT when expanding [60] and exhibits anti-inflammatory characteristics on the cross-talk between macrophages and adipocytes in mice [61].

Altogether our data suggest that treatment with dietary bio-actives during adipogenic differentiation, alters the cyto-/adipokine release towards a less pro-inflammatory secretion profile, indicated by an attenuated IL-6 and MCP-1 release. The combinations EPA/Res and Lyc/Res partially augmented the effects of the single compounds on the secretion of adiponectin, IL-6, MCP-1 and HGF, supporting the concept of strong amplifying/synergistic interactions between nutrients [62].

## Conclusions

Here we show for the first time, that the combination of the dietary bio-actives Lyc and EPA with Res might influence adipocyte function synergistically by affecting the balance between adipogenic, lipogenic and lipolytic gene expression, resulting in a reduced lipid accumulation and improved inflammatory profile. Our data suggest that applying combinations of bio-actives is a more favourable approach to tackle AT dysfunction because anti-lipogenic and anti-inflammatory effects can be magnified compared to the single dietary constituents [63–66]. Given that obesity is recognized as a state of chronic ‘low-grade inflammation’ [10, 11], reducing the secretion of pro- and stimulating anti-inflammatory adipokines with combinations of dietary bio-actives could contribute to manage and treat obesity-related metabolic complications.

## Additional files

Additional file 1: Table S1. Relevant donor characteristics from used human pre-adipocytes (HPAd). (DOCX 32 kb)
Additional file 2: Figure S1. Lipid droplet number, lipogenic gene expression markers and adipokine secretion of adipocytes treated with different doses of bio-active compounds. Assessment of different doses of Lyc, Res and EPA alone and of the combinations EPA/Res and Lyc/Res on distinct adipocyte parameters reflecting adipocyte function. Depicted are the effects on the number of lipid droplets (Lipid Droplets/Cell in % of Diff CTRL; A-B), the expression of the lipogenic genes LPL and GLUT-4 (FC ± error compared to Diff CTRL set as 1; C-D) and the secretion of the adipokines adiponectin and IL-6 (overall mean ± SEM; E-F) after 8 (C-F) or 14 (A-B) days treatment of HPAd (donors ≥ 2, n ≥ 2). These data indicate that not all parameters follow a linear dose-response relationship. (*) *p* < 0.05, (**) *p* < 0.01, (#) *p* < 0.001 (versus Diff CTRL, linear mixed model). (PDF 44 kb)
Additional file 3: Table S2. Altered Cellomics™ parameters for the analysis of HPAd (20x objective). (DOCX 33 kb)
Additional file 4: Table S3. Sequences of human primers and probes for adipocyte specific genes used for quantitative RT-PCR. (DOCX 34 kb)
Additional file 5: Detailed Statistical Analysis. (DOCX 27 kb)
Additional file 6: Figure S2. Effects of bio-active compounds on lipogenic/lipolytic gene expression markers and adipokine secretion in differentiated human adipocytes. Shown are the effects of the combinations EPA/Res and Lyc/Res on gene expression and adipokine secretion after 14 days treatment of HPAd. mRNA levels of lipogenesis markers LPL (A), GLUT-4 (B) and FAS (C) and the lipolytic marker PLIN-1 (D) were determined by quantitative RT-PCR and the accumulation of the adipokines adiponectin (E), IL-6 (F), MCP-1 (G) and PAI-1 (H) was measured in media after 5 days conditioning (day 10–14) on the LiquiChip® workstation. Data are shown as crude fold change (FC) ± error (based on SEM, A-D) compared to Diff CTRL set as 1 and as overall mean ± SEM (E-H) for one experimental series (all donors included: HPAd 1375, 1377 and super lot SL0035). (*) *p* < 0.05, (**) *p* < 0.01, (#) *p* < 0.001 (versus Diff CTRL, Student’s *t*-test)

## Abbreviations

Adipo: Adiponectin
AT: Adipose tissue
ATGL: Adipose triglyceride lipase
bFGF: basic fibroblast growth factor
C/EBP-alpha: CCAAT/enhancer binding protein alpha
DM: Differentiation medium
EPA: Eicosapentaenoic acid
FA: Fatty acid
FABP-4: Cytosolic fatty acid binding protein 4
FAS: Fatty acid synthase
FC: Fold change
FCS: Fetal calf serum
GLUT-4/SLC2A4: Glucose transporter type 4
GM: Growth medium
HFD: High fat diet
HGF: Hepatic growth factor
HPAd: Human pre-adipocytes
HSL: Hormone sensitive lipase
IL-6: Interleukin-6
IL-8: Interleukin-8
LD: Lipid droplet
LPL: Lipoprotein lipase
Lyc: (all-E)-lycopene
MCP-1/CCL2: Monocyte chemoattractant protein-1
n: number of experimental series
PAI-1: Plasminogen activator inhibitor
PBS: Phosphate-buffered saline
PLIN-1: Perilipin 1
PPAR-gamma: Peroxisome Proliferator-Activated Receptor Gamma
PUFA: Polyunsaturated fatty acid
Res: *trans*-resveratrol
SEM: Standard error of the mean
SGBS: Simpson-Golabi-Behmel syndrome
TAGs: Triacylglycerides.
