# Supplementary material for: Combinations of bio-active dietary constituents affect human white adipocyte function in-vitro
Source: Nutr Metab (Lond). 2016 Nov 21;13:84. doi: 10.1186/s12986-016-0143-5 (PMC5117626; doi:10.1186/s12986-016-0143-5)
Supplement: Additional file 1: Table S1. — Relevant donor characteristics from used human pre-adipocytes (HPAd). (DOCX 32 kb) [file 12986_2016_143_MOESM1_ESM.docx]

## Additional file 1:

## Table S1: Relevant donor characteristics from used human pre-adipocytes (HPAd)

| **Cells** | **Lot #** | ***ID*** | **Age** | **Gender** | **BMI** | **Number of donors** | **Ethnic group** | **Biopsy region** |
| --- | --- | --- | --- | --- | --- | --- | --- | --- |
| HPAd | 1375 | ***Donor 1*** | 41 | female | n.g. | 1 | Caucasian | abdominal, subcutaneous |
| HPAd | 1377 | ***Donor 2*** | 53 | female | n.g. | 1 | Caucasian | abdominal, subcutaneous |
| SL0035 (super lot) | 0035 | ***Donor Pool*** | 37- 57 | female | avg 27.9 | 5 | n.g. | Subcutaneous, abdominal (3) and gluteal (2) |

n.g. = not given
