# Supplementary material for: Combinations of bio-active dietary constituents affect human white adipocyte function in-vitro
Source: Nutr Metab (Lond). 2016 Nov 21;13:84. doi: 10.1186/s12986-016-0143-5 (PMC5117626; doi:10.1186/s12986-016-0143-5)

Lipid Droplet Quantification (ArrayScan) – Day 14

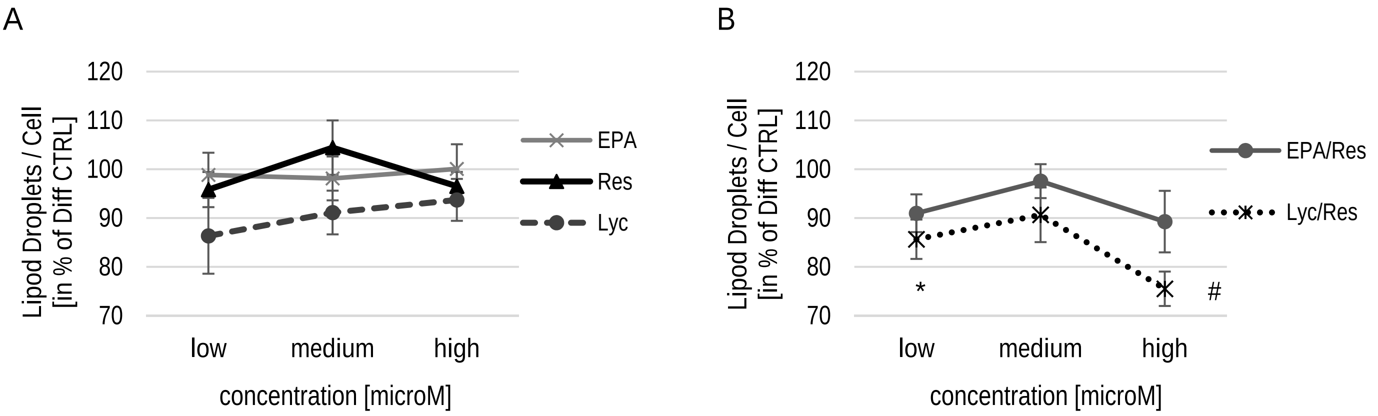

Gene Expression Analysis (TaqMan™) – Day 8

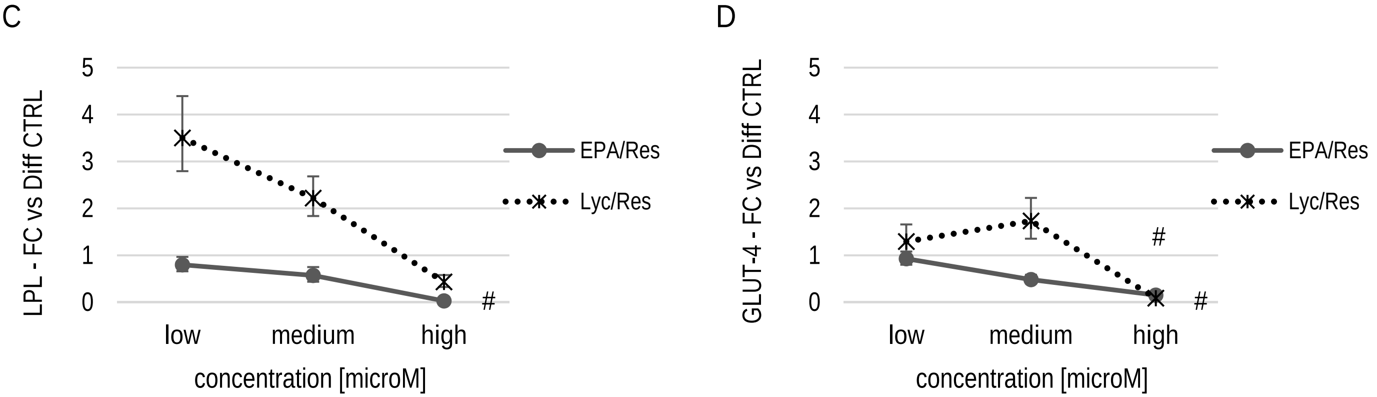

Adipo- and Cytokine Secretion (Luminex) – Day 8

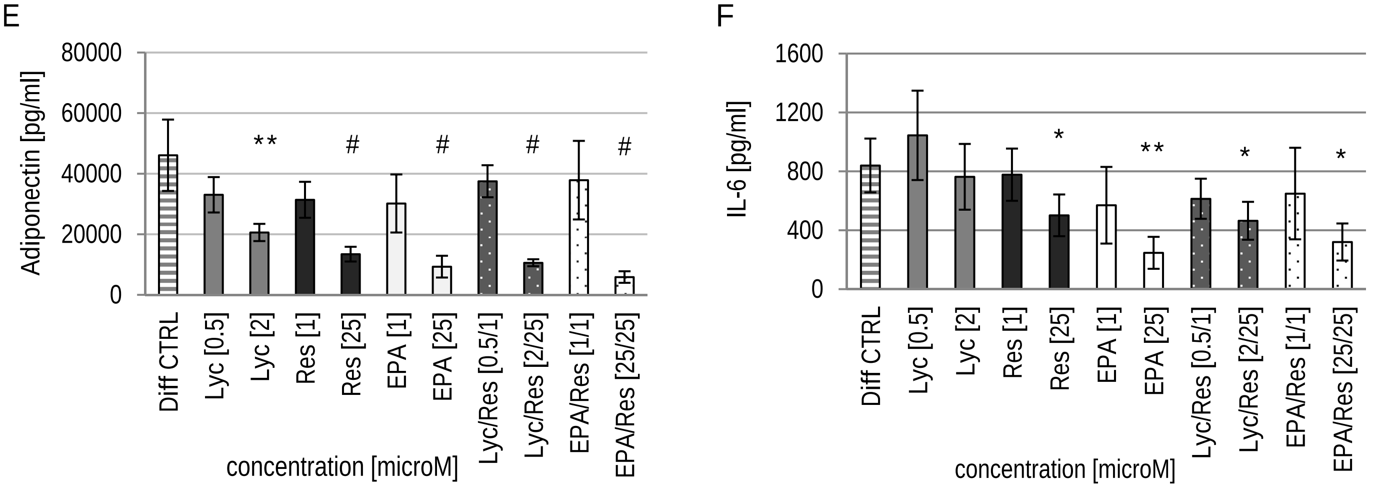

Supplement: Additional file 2: Figure S1. — Lipid droplet number, lipogenic gene expression markers and adipokine secretion of adipocytes treated with different doses of bio-active compounds. Assessment of different doses of Lyc, Res and EPA alone and of the combinations EPA/Res and Lyc/Res on distinct adipocyte parameters reflecting adipocyte function. Depicted are the effects on the number of lipid droplets (Lipid Droplets/Cell in % of Diff CTRL; A-B), the expression of the lipogenic genes LPL and GLUT-4 (FC ± error compared to Diff CTRL set as 1; C-D) and the secretion of the adipokines adiponectin and IL-6 (overall mean ± SEM; E-F) after 8 (C-F) or 14 (A-B) days treatment of HPAd (donors ≥ 2, n ≥ 2). These data indicate that not all parameters follow a linear dose-response relationship. (*) p < 0.05, (**) p < 0.01, (#) p < 0.001 (versus Diff CTRL, linear mixed model). (PDF 44 kb) [file 12986_2016_143_MOESM2_ESM.pdf]
