# Supplementary material for: Combinations of bio-active dietary constituents affect human white adipocyte function in-vitro
Source: Nutr Metab (Lond). 2016 Nov 21;13:84. doi: 10.1186/s12986-016-0143-5 (PMC5117626; doi:10.1186/s12986-016-0143-5)
Supplement: Additional file 3: Table S2. — Altered Cellomics™ parameters for the analysis of HPAd (20x objective). (DOCX 33 kb) [file 12986_2016_143_MOESM3_ESM.docx]

## Additional file 2:

## Table S2: Altered Cellomics™ parameters for the analysis of HPAd (20x objective)

| Object Identification |  | |  |  | |
| --- | --- | --- | --- | --- | --- |
| **Channel 1: Objects** Method: | FixedThreshold | | **Channel 2: Spots** Method: | TriangThreshold | |
| Value: | 125 | | Value: | 0.499 | |
| Object Selection Parameter | Min | Max |  | Min | Max |
| **Channel 1: Objects**  ObjectAreaCh1: | 50 | 10000 | **Channel 2: Spots**  SpotAreaCh2: | 3 | 10000 |
| ObjectAvgIntenCh1: | 0 | 4095 | SpotAvgIntenCh2: | 10 | 4095 |
| ObjectTotalIntenCh1: | 0 | 4411431044 | SpotTotalIntenCh2: | 500 | 4411431044 |
| Assay Parameters |  | |  |  | |
| BackgroundCorrectionCh1: | 140 | | BackgroundCorrectionCh2: | 140 | |
| ObjectSegmentationCh1: | 20 | | CircModifierCh2 | 127 | |
| SmoothFactorCh1: | 5 | | RingDistanceCh2: | -512 | |
| SmoothMethodCh1: | 1 | | SpotDetectRadiusCh2: | 5 | |
| RejectBorderObjectsCh1: | 0 | | SpotSmoothFactorCh2: | 1 | |
| PixelSize: | 1.024 microns | | SpotSmoothMethodCh2: | 1 | |

Displayed values are pixel number. ThermoFisher Bioapplication applied: SpotDetector^®^
