# Supplementary material for: Combinations of bio-active dietary constituents affect human white adipocyte function in-vitro
Source: Nutr Metab (Lond). 2016 Nov 21;13:84. doi: 10.1186/s12986-016-0143-5 (PMC5117626; doi:10.1186/s12986-016-0143-5)
Supplement: Additional file 4: Table S3. — Sequences of human primers and probes for adipocyte specific genes used for quantitative RT-PCR. (DOCX 34 kb) [file 12986_2016_143_MOESM4_ESM.docx]

## Additional file 3:

## Table S3: Sequences of human primers and probes for adipocyte specific genes used for quantitative RT-PCR

| **Gene name** | **Forward Primer**  **Reverse Primer** | **Probe**  (FAM conjugated) |
| --- | --- | --- |
| **Peroxisome proliferator activated receptor gamma 1+2 (PPARγ 1+2)** [NM_138712.3](http://www.ncbi.nlm.nih.gov/nucleotide/116284369?report=genbank&log$=nucltop&blast_rank=8&RID=KZ5T3EY3014), [NM_015869.4](http://www.ncbi.nlm.nih.gov/nucleotide/116284371?report=genbank&log$=nucltop&blast_rank=7&RID=KZ5T3EY3014) | 5`TCAGAAATGCCTTGCAGTG`3  5`TTCTCGGCCTGTGGCATC`3 | 5`TGTCTCATAATGCCATCAGGTTTGGGC`3 |
| **CCAAT/enhancer binding protein alpha (C/EBPα)** [NM_001287435.1](http://www.ncbi.nlm.nih.gov/nucleotide/566559993?report=genbank&log$=nucltop&blast_rank=1&RID=M1UD78GX014) | 5`AAGAAGTCGGTGGACAAGAACAG`3  5`TGCGCACCGCGATGT`3 | 5`AACGAGTACCGGGTGCGGCG`3 |
| **Lipoprotein lipase (LPL)** [NM_000237.2](http://www.ncbi.nlm.nih.gov/nucleotide/145275217?report=genbank&log$=nucltop&blast_rank=1&RID=KXKJ4AR6014) | 5`CAGCAAAACCTTCATGGTGAT`3  5`CAAGTTTTGGCACCCAACTC`3 | 5`CATACATTCCTGTTACCGTCCAGCCA`3 |
| **Fatty acid synthase (FAS)** [NM_004104.4](http://www.ncbi.nlm.nih.gov/nucleotide/41872630?report=genbank&log$=nucltop&blast_rank=2&RID=KXY3C6JZ01R) | 5`CACCTATGGCCTGCAGTGC`3  5`TGATGCAGTCGATGTAGTAGGCA`3 | 5`CCCGAGCTGCGCCCCTTG`3 |
| **Fatty acid binding protein 4 (FABP-4)** [NM_001442.2](http://www.ncbi.nlm.nih.gov/nucleotide/168480125?report=genbank&log$=nucltop&blast_rank=1&RID=M205BNH701R) | 5`AAATGTGTGATGCTTTTGTAGGTACC`3  5`CCATGCCAGCCACTTTCC`3 | 5`ATGAAAGAAGTAGGAGTGGGCTTTGCCACC`3 |
| **Glucose transporter 4 (GLUT-4)** [NM_001042.2](http://www.ncbi.nlm.nih.gov/nucleotide/83722278?report=genbank&log$=nucltop&blast_rank=1&RID=KZ74MMCA01R) | 5`CCGCTACCTCTACATCATCCAGA`3  5`CAGAAACATCGGCCCAGC`3 | 5`CCTGCCAGAAAGAGTCTGAAGCGCC`3 |
| **Adipose triglyceride lipase (ATGL)** [NM_020376.3](http://www.ncbi.nlm.nih.gov/nucleotide/300796746?report=genbank&log$=nucltop&blast_rank=3&RID=KZ565WA601R) | 5`CGAGAATGTCATTATATCCCACTTCA`3  5`TGAGCCCACAGTACACGGG`3 | 5`AAGGACGAGCTCATCCAGGCCAATG`3 |
| **Hormone sensitive lipase (HSL/ LIPE)** [NM_005357.3](http://www.ncbi.nlm.nih.gov/nucleotide/542133076?report=genbank&log$=nucltop&blast_rank=7&RID=M1KSR5YB01R) | 5`CTGCATAAGGGATGCTTCTATC`3  5`CCTGTCTCGTTGCGTTTGTAGTA`3 | 5`CTGCCTGGGCTTCCAGTTCACGC`3 |
| **Perilipin 1 (PLIN-1)** NM_001145311.1 | 5`CCCCCTGAAAAGATTGCTTCT`3  5`GGAACGCTGATGCTGTTTCTG`3 | 5`CCATCTCTCCACCCGCCTCCGC`3 |
| **Endogenous control** | **Forward Primer**  **Reverse Primer** | **Probe**  (VIC conjugated) |
| **18S rRNA**  [NR_003286.2](http://www.ncbi.nlm.nih.gov/nucleotide/225637497?report=genbank&log$=nucltop&blast_rank=2&RID=KZ704TPK01R) | 5`CGGCTACCACATCCAAGG`3  5`CGGGTCGGGAGTGGGT`3 | 5`TTGCGCGCCTGCTGCCT`3 |
