# Supplementary material for: Combinations of bio-active dietary constituents affect human white adipocyte function in-vitro
Source: Nutr Metab (Lond). 2016 Nov 21;13:84. doi: 10.1186/s12986-016-0143-5 (PMC5117626; doi:10.1186/s12986-016-0143-5)
Supplement: Additional file 5: — Detailed Statistical Analysis. (DOCX 27 kb) [file 12986_2016_143_MOESM5_ESM.docx]

## Detailed Statistical Analysis

Statistical programming was performed with the statistical software R [1, version 3.0.2]. Additional packages used for analysis were multcomp [2, version 1.3-6] and nlme [3, version 3.1-117].

**Analysis of Array-Scan data:**

For standardisation purposes, each 24-well plate contained a Diff CTRL (triplicates) and all treatment triplicates were calculated in % of the Diff CTRL average per plate.

All parameters were investigated for normality of distribution (visually and using the Shapiro Wilk test). A log-transformation (base 10) was performed to correct for observed skewness of the distribution of the data. Analysis then took place with a hierarchical linear mixed model. We included donor, treatment and donor-treatment interaction as fixed effect and additionally allowed different variances per donor/treatment stratum, since visual inspection showed that variance heterogeneity between strata had remained even after log-transformation. To ensure that the assumptions of the used model were met we inspected goodness of fit plots.

The hierarchy of the experiment was reflected by using nested random effects (replicate nested in plate nested in passage number nested in experiment). Donor differences per treatment and overall and treatment differences (vs. Diff CTRL) per donor and averaged across donors were tested. p-values were adjusted for multiple comparisons for overall averages [2].

**Analysis of** ΔΔ**CT-values for gene expression data:**

mRNA abundance was calculated using the comparative C_T_ method: ΔC_T_ = C_T_ [*gene of interest*] – C_T_ [*endogenous control*] and ΔΔC_T_ = ΔC_T_ [*Diff CTRL cells*] - ΔC_T_ [*treated cells*]. ΔΔC_T_ values were calculated using the Diff CTRL cells per plate to standardize the data points. A hierarchical linear mixed model was used to estimate the mean ΔΔCT across all experiments and donors. For this, we included treatment as fixed effect and additionally allowed different variances per donor/treatment stratum, since visual inspection showed variance heterogeneity between strata. The hierarchy of the experiment was reflected by using nested random effects (replicate nested in well nested in plate nested in donor). The estimated mean ΔΔCT per treatment together with its estimated standard deviation were then used to calculate 2^(-ΔΔCT) together with its range (+/- error based on SEM). To ensure that the assumptions of the used model were met we inspected goodness of fit plots.

**Analysis for adipokines in supernatants:**

All parameters were investigated for normality of distribution (visually and by using the Shapiro Wilk test). A log-transformation (base 10) was performed to correct for observed skewness of the distribution of the data. Analysis then took place similar to before with a hierarchical linear mixed model. We included donor, treatment and donor-treatment interaction as fixed effect and additionally allowed different variances per donor/treatment stratum, since visual inspection showed that variance heterogeneity between strata had remained even after log-transformation.

The hierarchy of the experiment was reflected by using nested random effects (replicate nested in well nested in plate nested in experiment). Donor differences per treatment and overall and treatment differences (vs. Diff CTRL) per donor and averaged across donors were tested. Univariate and adjusted p-values were reported. To ensure that the assumptions of the used model were met we inspected goodness of fit plots.

**Reference:**

1. R Core Team (2013). R: **A language and environment for statistical computing**. R Foundation for Statistical Computing, Vienna, Austria.
2. Torsten Hothorn, Frank Bretz and Peter Westfall (2008). **Simultaneous Inference in General Parametric Models**. Biometrical Journal 50(3), 346--363.
3. Pinheiro J, Bates D, DebRoy S, Sarkar D and R Core Team (2014). nlme: Linear and Nonlinear Mixed Effects Models. R package version 3.1-117
