# Supplementary figures and images for: Combinations of bio-active dietary constituents affect human white adipocyte function in-vitro
Source: Nutr Metab (Lond). 2016 Nov 21;13:84. doi: 10.1186/s12986-016-0143-5 (PMC5117626; doi:10.1186/s12986-016-0143-5)

# Gene Expression Analysis (TaqMan™) – Day 14

A

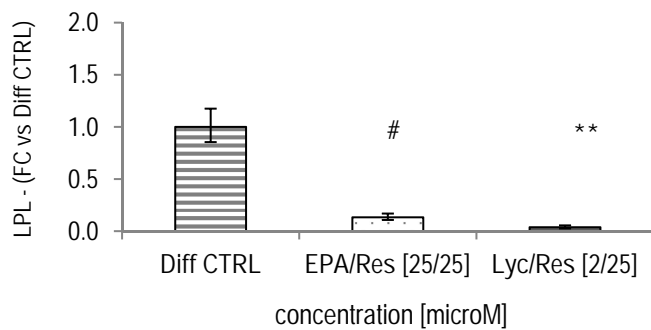

B

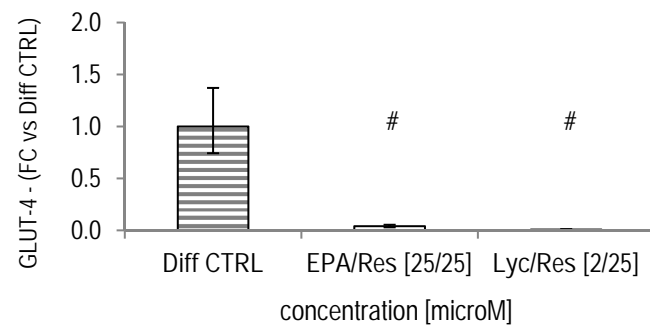

C

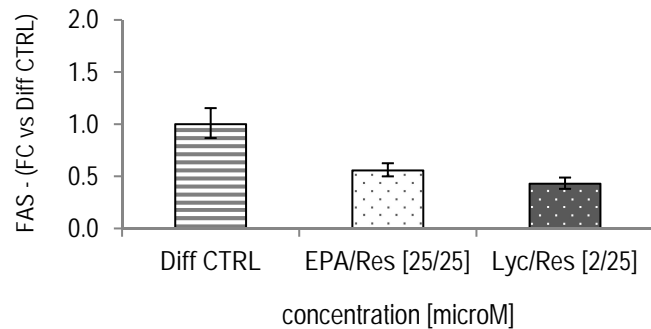

D

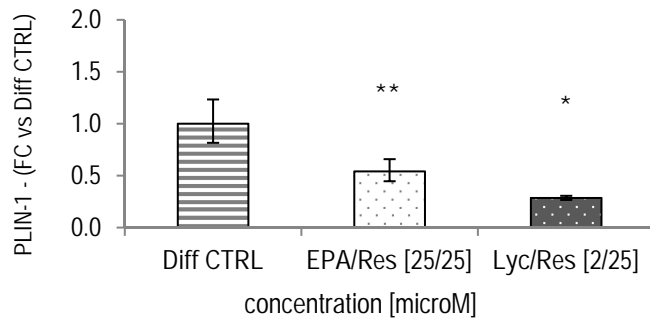

## Adip- and Cytokine Secretion (Luminex) – Day 14

E

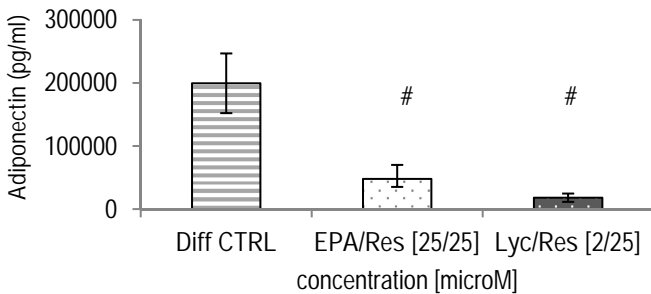

F

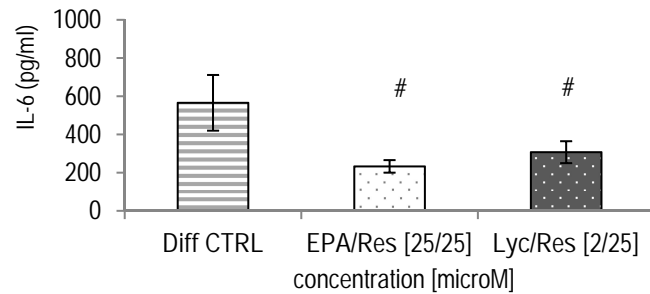

G

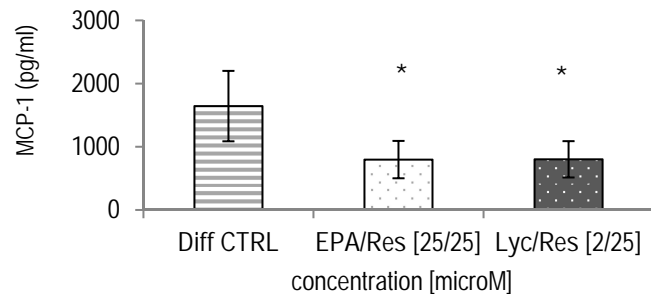

H

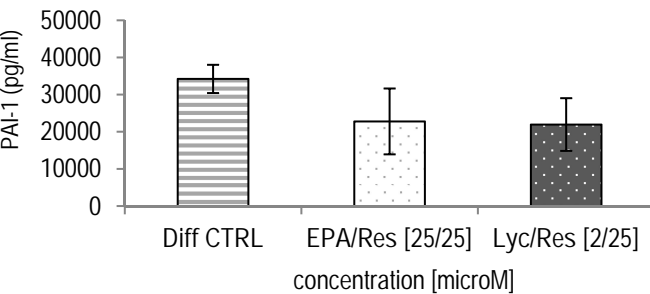

Supplement: Additional file 6: Figure S2. — Effects of bio-active compounds on lipogenic/lipolytic gene expression markers and adipokine secretion in differentiated human adipocytes. Shown are the effects of the combinations EPA/Res and Lyc/Res on gene expression and adipokine secretion after 14 days treatment of HPAd. mRNA levels of lipogenesis markers LPL (A), GLUT-4 (B) and FAS (C) and the lipolytic marker PLIN-1 (D) were determined by quantitative RT-PCR and the accumulation of the adipokines adiponectin (E), IL-6 (F), MCP-1 (G) and PAI-1 (H) was measured in media after 5 days conditioning (day 10–14) on the LiquiChip® workstation. Data are shown as crude fold change (FC) ± error (based on SEM, A-D) compared to Diff CTRL set as 1 and as overall mean ± SEM (E-H) for one experimental series (all donors included: HPAd 1375, 1377 and super lot SL0035). (*) p < 0.05, (**) p < 0.01, (#) p < 0.001 (versus Diff CTRL, Student’s t-test) [file 12986_2016_143_MOESM6_ESM.pdf]
